# Early Experience With a Novel Super-Hydrophilic Laparoscopic Scope Cleaning Device and Narrative Review of Available Cleaning Strategies

**DOI:** 10.1177/15533506241281316

**Published:** 2024-09-01

**Authors:** Graham J. Spurzem, Ryan C. Broderick, Patricia R. Cota, Bryan J. Sandler, Garth R. Jacobsen, Santiago Horgan

**Affiliations:** 1 Department of Surgery, Division of Minimally Invasive Surgery, 8784 University of California San Diego, San Diego, CA, USA

**Keywords:** laparoscopic lens fogging, hydrophilic, cold plasma, laparoscope cleaning

## Abstract

**Background:**

Impaired visibility is a challenge in laparoscopic surgery. Frequent scope removal increases operative time, reduces efficiency, and potentially compromises patient safety. We examine our initial experience with a novel cleaning device that applies cold plasma to the scope lens and review current available laparoscope cleaning methods.

**Methods:**

The novel device was used in a variety of laparoscopic general surgery cases from April to November 2023. Primary outcome was number of scope removals per case. Secondary outcomes were time spent cleaning and number of times the scope became smudged or dirty with blood/tissue debris (debris events). An existing device that utilizes heated anti-fogging solution was used for comparison.

**Results:**

97 cases were included (31 with novel device and 66 with existing device). Scope removal rate for the novel device was lower compared to the existing device (0.87 ± 1.02 vs 0.97 ± 1.20 removals/case, *P* = 0.69), but not statistically significant. Average number of debris events was also lower for the novel device, but not statistically significant (0.90 ± 0.94 vs 1.0 ± 1.18 debris events/case, *P* = 0.69). Average total time spent cleaning per case was similar between devices (16.9 ± 24.0 vs 15.9 ± 18.7 seconds, *P* = 0.82).

**Conclusion:**

This study demonstrates that a hydrophilic scope cleaning device has comparable performance to heated anti-fogging solution and may reduce scope removals and debris events. Direct comparisons between cleaning products are lacking. Surgeons are most likely to be successful with the cleaning strategy that best suits one’s surgical practice.

## Introduction

Since its introduction in the late 1980s, laparoscopic surgery has gained widespread adoption and become the standard of care for many surgical procedures.^1^ Despite decades of experience with laparoscopy, significant technological challenges remain, including the management of an impaired visual field. Condensation on the scope lens, also known as laparoscopic lens fogging (LLF), due to differences in temperature and humidity between the operating room and abdominal cavity is a frequent challenge.^2^ Blood, other intra-abdominal fluid, tissue debris, and smoke from tissue coagulation can also smudge the lens and necessitate scope removal from the abdomen for cleaning.^3^ Observational studies have demonstrated that over 1 third of laparoscopic operating time is spent with impaired visualization.^4^ Frequent scope removal increases operative time, reduces surgeon efficiency, and can potentially compromise patient safety.^5^

Various strategies have been developed to preserve lens clarity and reduce the need for scope removal. Initial approaches focused on warming the scope lens prior to use and the development of anti-fogging solution, which is widely used today.^6^ Technical modifications to the laparoscope that enable intracorporeal lens cleaning have also been translated into commercial products.^6,7^ There is no consensus on the most effective and efficient method for laparoscopic lens cleaning during minimally invasive abdominopelvic surgery, although it remains a vexing problem. A recently developed product offers a unique approach by attempting to prevent LLF altogether to reduce scope removals. The Plasma Shield® device by Plasmatica (Moshava, Israel) applies cold plasma to the scope lens to create an activated super-hydrophilic surface, which promotes the fusion of individual liquid droplets into a thin, transparent liquid film. This film then protects the scope lens from fogging and other operative debris. To the best of our knowledge, there is no literature available on the performance of this device or its ability to reduce scope removal during surgery. We examine the performance of this novel device in comparison to heated anti-fogging solution during a range of minimally invasive operations and review other available cleaning strategies and devices.

## Materials and Methods

The novel device was used at our academic center in a variety of foregut, bariatric, hepatobiliary, and general surgery cases between April and November 2023. Before each operation, the surgical nurse or technologist was educated on the proper use of the device. The device was used with both 5 mm and 10 mm laparoscopes on the Stryker 1688 AIM 4K platform (Kalamazoo, MI, USA). During each case, the camera was operated by a minimally invasive surgery (MIS) fellow and the operation was performed by an experienced MIS surgeon. This study was approved by the University of California, San Diego Institutional Review Board.

Device performance was evaluated during each individual procedure. The primary outcome measure was number of scope removals per case. Secondary outcomes were total time spent cleaning the scope and number of times the scope became smudged or dirty with blood or tissue debris (debris events). In using the novel device, true lens fogging was not observed; however, lens smudging was a common occurrence, so this was chosen as an outcome measure. For comparison, these same metrics were measured during similar cases using the Clearify™ Visualization System (Covidien, Mansfield, MA, USA), which applies heated anti-fogging solution to the scope lens. This device was chosen for comparison because it is widely used, has a similar price as the novel device, and is the current standard of care in our hospital system. Clearify was used according to the manufacturer directions. Scope cleaning time was measured during surgery from the time of scope removal to reinsertion after cleaning. The initial application of cold plasma occurred prior to the start of each case and was not included in overall cleaning time. All statistical analyses were performed in R (Version 4.1.2, Vienna, Austria). Fisher’s exact test was used for categorical variables and Student’s *t* test was used for continuous variables. A *P*-value of <0.05 was considered statistically significant.

**Figure 1.**
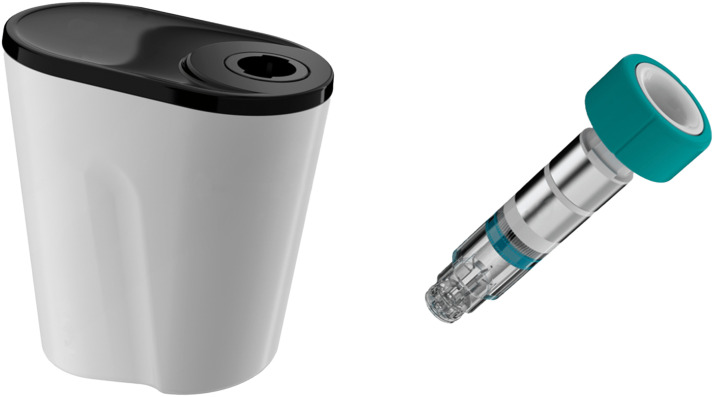
Novel laparoscopic scope cleaning system comprised of a reusable base (left) and disposable scope chamber (right) that fits into the base.

**Figure 2.**
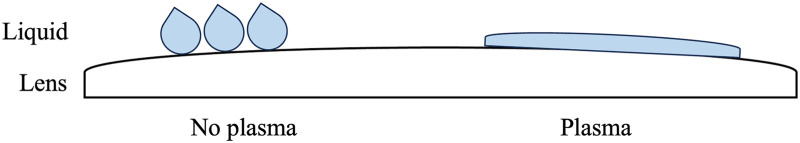
Application of cold plasma to the laparoscope lens promotes fusion of individual liquid droplets into a thin, transparent film.

The novel system is comprised of a reusable base and a disposable scope chamber that is integrated into a sterile surgical drape designed to completely cover the base (Figure 1). The system also includes a bottle of liquid to be applied to 1 side of a small sponge. The chamber is first inserted into the reusable base, then after draping and an on-screen prompt, the scope is inserted into the chamber for 10 seconds to apply cold plasma to the lens. The activated scope lens is then touched to a wet area of the sponge, followed by a dry area to remove excess liquid. The scope is then ready for use. This process is designed to fuse individual liquid droplets into a thin, transparent film on the scope lens (Figure 2).

## Results

A total of 31 cases using the novel device and 66 cases using the existing device were included. The 2 devices were used in a similar assortment of laparoscopic operations (Table 1). The devices were both most commonly used during laparoscopic sleeve gastrectomy, which comprised 45.2% of cases for the novel device and 34.8% for the existing device.

**Table 1.** List of Laparoscopic Operations Completed With Each Cleaning Device.

	Number of Cases, n (%)	
Operation	Plasma Shield (N = 31)	Clearify (N = 66)
Laparoscopic sleeve gastrectomy	14 (45.2)	23 (34.8)
Laparoscopic cholecystectomy	6 (19.4)	10 (15.2)
Laparoscopic hiatal hernia repair	3 (9.7)	21 (31.8)
Laparoscopic inguinal/ventral hernia repair	3 (9.7)	1 (1.5)
Diagnostic laparoscopy	2 (6.5)	5 (7.6)
Laparoscopic adrenalectomy	1 (3.2)	1 (1.5)
Laparoscopic hepaticojejunostomy	1 (3.2)	-
Laparoscopic gastric lipoma resection	1 (3.2)	-
Laparoscopic fundoplication takedown	-	3 (4.5)
Laparoscopic esophageal myotomy	-	1 (1.5)
Laparoscopic gastric pacemaker replacement	-	1 (1.5)

Device performance was first evaluated based on the average number of intraoperative events per case. The scope removal rate for the novel device was slightly lower compared to the existing device (novel: 0.87 ± 1.02 vs existing: 0.97 ± 1.20 removals/case, *P* = 0.69), but not statistically significant. The average total time spent cleaning the scope per case was similar between devices (novel: 16.9 ± 24.0 vs existing: 15.9 ± 18.7 seconds, *P* = 0.82). The average number of debris events was slightly lower for the novel device, but also not statistically significant (novel: 0.90 ± 0.94 vs existing: 1.0 ± 1.18 debris events/case, *P* = 0.69). A summary of these results is detailed in Table 2.

**Table 2.** Comparison of Laparoscopic Scope Cleaning Device Performance Based on the Average Number of Intraoperative Events per Case.

	Mean (SD) per Case		
Intraoperative Event	Plasma Shield (N = 31)	Clearify (N = 66)	*P*-value
Scope removal (n)	0.87 (1.02)	0.97 (1.20)	0.69
Debris (n)	0.90 (0.94)	1.0 (1.18)	0.69
Time spent cleaning (sec)	16.9 (24.0)	15.9 (18.7)	0.82

Device performance was then analyzed based on the proportion of cases involving a finite number of intraoperative events. The proportion of cases requiring no scope removals (novel: 45.2% vs existing: 42.4%, *P* = 0.83), 1 scope removal (32.3% vs 34.8%, *P* = 0.99), and 2 scope removals (16.1% vs 13.6%, *P* = 0.76) was similar between the devices. The novel and existing devices had a similar proportion of cases with zero debris events (novel: 38.7% vs existing: 39.4%, *P* = 0.99), 1 debris event (38.7% vs 36.4%, *P* = 0.99), and 2 debris events (19.4% vs 16.7%, *P* = 0.97). Of note, the existing device had 3 cases with 3 total debris events and 2 cases with greater than 3 debris events, compared to zero cases for the novel device. A summary of these results is detailed in Table 3.

**Table 3.** Comparison of Laparoscopic Scope Cleaning Device Performance Based on the Proportion of Cases Involving a Finite Number of Intraoperative Events.

	Number of Cases, n (%)		
Number of Scope Removals	Plasma Shield (N = 31)	Clearify (N = 66)	*P*-value
0	14 (45.2)	28 (42.4)	0.83
1	10 (32.3)	23 (34.8)	0.99
2	5 (16.1)	9 (13.6)	0.76
3	1 (3.2)	4 (6.1)	-
>3	1 (3.2)	2 (3.0)	-
Number of debris events			
0	12 (38.7)	26 (39.4)	0.99
1	12 (38.7)	24 (36.4)	0.99
2	6 (19.4)	11 (16.7)	0.97
3	0 (0.0)	3 (4.5)	-
>3	0 (0.0)	2 (3.0)	-

## Discussion

In this study, we examine our initial experience with a novel laparoscopic scope cleaning device that relies on the physical properties of cold plasma to create a hydrophilic surface on the scope lens, preventing liquid droplet formation and lens fogging. We compare the performance of this device during a variety of laparoscopic procedures to another widely used cleaning device that utilizes heated anti-fogging solution. This study is the first to assess the performance of this novel cleaning device and offers insight into its subtleties and potential best use cases.

We found that the device does effectively prevent lens fogging and slightly reduces scope removals and debris events compared to heated anti-fogging solution, with differences that could potentially trend toward statistical significance in a larger sample size. Interestingly, in using the novel device we did not observe any instances of true LLF. Instead, we found that following application of cold plasma and introduction of the laparoscope into the abdomen, the visual field would often appear smudged and obscure our view. This could sometimes be remedied by rubbing the lens on a nearby intra-abdominal organ (eg, liver or small bowel), but in several instances the scope ultimately required removal for cleaning and reapplication of liquid on the lens to recreate the protective aqueous film. Despite this, it is possible that the novel device’s ability to reduce scope removals and protect against debris events afforded a larger percentage of laparoscopic operative time with clear visibility, potentially improving operative efficiency. Further study of this device in a wider array of clinical scenarios and an assessment of surgeon satisfaction would also be helpful to assess its clinical performance more completely. Overall, the device performed well compared to our standard scope cleaning method and it is relatively simple to use and operate.

A major drawback of this device relative to other cleaning devices is the lack of intracorporeal lens cleaning capability. While the thin liquid film that forms on the activated hydrophilic lens may provide some protection, any significant insult to the lens from either blood or operative debris most often required removal for cleaning. While the lens can be quickly and easily cleaned intracorporeally with saline irrigation, this requires the use of an additional irrigation device, which is inefficient and not cost effective if irrigation is not already opened for use. The addition of a laparoscope attachment that delivers a small saline wash over the scope lens as needed would be a helpful adjunct. With this limitation in mind, the device is probably best suited for operations with an inherently low risk of insult to the scope lens, that is those cases with a low likelihood of bleeding and limited use of tissue coagulation. In the absence of this, the device performs well in terms of preventing LLF and maintaining clear visibility. The device may also be especially useful during robotic procedures, as scope removal and cleaning takes a considerable amount of time and interrupts operative flow.

As with other operating room innovations, cost-effectiveness is often a concern when implementing new technology. The reusable base of the novel device is provided to hospitals free of charge, and the disposable chamber costs approximately $60 per unit. The cost per unit of the heated anti-fogging device used for comparison is $58.54 in our hospital system. The comparable cost profile of this novel device in relation to a direct competitor opens the door for future potential cost savings, for example by reducing operative time. It’s possible that scope removal rates may be further reduced with increased device familiarity and surgeon progression along the learning curve. Further studies are needed however to evaluate the impact of this novel system on surgeon operative efficiency and patient outcomes.

Our study has several limitations, including its single-center design and device use limited to operations performed by minimally invasive general surgeons. The propensity of the scope to become dirty during any given case is also dependent on the camera operator to a degree. We did not include an analysis of operative time, which is a helpful adjunct to assess operative efficiency. In addition, we did not compare performance of the novel device to any other commonly used methods or commercially available cleaning devices apart from Clearify. Further study is also needed to determine how long a single application of cold plasma can maintain the hydrophilic surface and how often during longer operations the plasma must be reapplied to the scope lens to maintain clear visibility.

## Review of Available Cleaning Strategies

The problem of impaired visibility and LLF has plagued laparoscopic surgery since its inception. LLF not only harms visibility and induces surgeon frustration, but it also increases operative time and may increase the likelihood of intraoperative complications.^3,5,8^ Several scope cleaning methods have been developed and can be divided into 3 broad categories: (1) laparoscope heating, (2) anti-fogging surfactant solutions, and (3) commercial cleaning devices. Here we provide a narrative review of the evidence pertaining to each cleaning strategy and explore the impact of these strategies on available technical and clinical outcomes.

### Laparoscope Heating

Following introduction of a laparoscope into the warm, moist intra-abdominal cavity, condensation occurs on the lens largely due to the temperature difference between the laparoscope and patient. Multiple studies have demonstrated that preheating the laparoscope prior to use can prevent LLF.^9-12^ Van Deurzen et al. reported a simple and cost-effective method for preventing LLF by lens heating using a sterilized thermos flask filled with hot sterile water.^2^ Similarly, Brown et al. utilized a water bath capable of maintaining hot water at a stable 120°F to warm the scope during surgery, and anecdotally reported significant reductions in lens fogging events during a 5-year observation period.^13^ However, it is obvious that the benefit of preheating the laparoscope diminishes as the scope cools, resulting in condensation and need for scope removal to clean and reheat. This has led many to postulate that anti-fogging solutions may be more effective due to the physical properties of surfactant, but this has been disputed by several investigations discussed in the following section.^9-11,14^

### Anti-fogging Surfactant Solutions

Anti-fogging surfactant solutions are applied directly to the scope lens, lowering the surface tension of liquid droplets to prevent condensation. Several products exist, including FRED^TM^ (85% water, <15% isopropyl alcohol, <2% surfactant), Ultra-Stop^TM^ (ethanol, purified water, <5% surface-active substances), Resoclear® sterile wipes, chlorhexidine, and povidone-iodine (betadine) solutions, each with varying effectiveness in the literature compared to different experimental controls. In an experimental abdominal model, Manning et al. compared several anti-fogging techniques including scope warmers, FRED, Resoclear, chlorhexidine, betadine, and scope immersion in heated saline.^12^ They found that all products had some degree of visibility benefit, but FRED performed the best, while betadine and Resoclear were no better than a scope warmer. In contrast, multiple studies have demonstrated an apparent superiority of heated sterile water to anti-fogging solutions. In a randomized, double-blind, controlled trial of patients undergoing laparoscopic gynecologic surgery, Song and Lee compared warm saline, Ultra-Stop, and chlorhexidine solution on a 10-point visual clarity scale to assess the severity of LLF during the first 3 minutes after laparoscope insertion into the abdomen.^11^ They found that warm saline led to significantly fewer fogging events than anti-fogging or chlorhexidine solutions. Similarly, Merkx et al. reported that heated sterile water caused significantly fewer fogging events than ResoClear wipes during laparoscopic donor nephrectomy.^10^ In a series of 40 laparoscopic urology procedures, Drysch et al. found that a heated water bath produced fewer fogging events than Clearify.^9^ While others in the literature support the efficacy of surfactant solutions, rigorous quantitative analyses are lacking.^15^ With no consensus on the optimal method to clean the laparoscope and prevent LLF, many inventors have developed commercial products in response.

### Commercial Cleaning Devices

A number of products have been developed with the goal of preventing LLF and/or enabling intracorporeal lens cleaning to clear operative debris. EndoClear (Virtual Ports Ltd, Misgav, Israel) is a device comprised of 2 cleaning fabric leaflets designed to be clipped to the abdominal wall at the start of an operation. The laparoscope can then be cleaned on the fabric implanted inside the abdomen. In a series of 40 laparoscopic fundoplications using EndoClear, Cassera et al. reported a 1.7-minute reduction in mean scope cleaning time with the device compared to anti-fogging solution.^16^ Other devices attach to the laparoscope itself to support lens cleaning. ClearScope^TM^ developed by ClearCam (Austin, TX, USA) is an external sleeve attachment compatible with 5 mm scopes that acts as a windshield wiper for the laparoscope lens. The developers of this device conducted a study in 2022 detailing a series of 167 cases and found that the device eliminated the need for scope removal in 90.14% of debris events with good surgeon satisfaction.^7^ MedeonBio (Sunnyvale, CA, USA) promotes ClickClean^TM^, a device that sheaths the laparoscope and covers the lens with a thin exchangeable biofilm.^17^ When the film is soiled with blood or tissue debris, clicking a button on the device advances a new clean film over the lens, restoring a clear visual field. The FloShield system® (Cupertino, CA, USA), compatible with 5 mm, 10 mm, and robotic laparoscopes, takes a different approach by shielding the laparoscope lens with a continuous flow of carbon dioxide (CO_2_) gas. The system includes a sheath that diverts a portion of the CO_2_ from existing insufflation tubing to the tip of the laparoscope, protecting the lens from condensation, debris and smoke.^18^ A modification to the system, called Flo-X in situ, allows surgeons to flush the lens with a burst of surfactant solution as desired. A single center randomized prospective study by Bendifallah et al. compared FloShield to a water/betadine solution during gynecologic cases and found that use of FloShield resulted in significantly fewer mean scope removals per case (2.8 vs 7.0 removals, *P* < 0.001).^19^ OpClear® (Cipher Surgical Ltd., Coventry, UK) leverages a similar concept with 2 main features: maintenance of continuous visual clarity with intelligent CO_2_ flow at the laparoscope tip and on-demand CO_2_ and saline lens washes to eliminate fogging and operative debris. A small series of 15 colorectal cases with this device reported no need for scope removal when vision was impaired during surgery.^20^ A randomized controlled trial comparing OpClear and warm saline during colorectal cases found that OpClear reduced the subjective physical demand of surgeons by reducing the number of lens washes required outside of the abdomen.^21^ To date, there have been no direct comparisons of any of these cleaning devices to each other.

### Outcomes

Among easily accessible methods, heating the laparoscope lens with sterile fluid and FRED anti-fogging solution have the most evidence supporting their effectiveness in terms of improving visibility. Commercial cleaning devices were universally more effective than experimental controls in the few available studies discussed in the previous section. However, despite the reported efficacy of these various products, most of the experimental evidence has focused on outcomes related to improving operative visibility, rather than clinical outcomes such as operative time or complication rates. Of the available published data, no device or method has conferred any consistent measurable clinical benefit.

A small number of studies have attempted to quantify the clinical impact of scope removal due to an impaired visual field. Abbit et al. investigated the amount of time a laparoscope is withdrawn for cleaning during surgery and found a significant correlation between increased scope removal and increased estimated blood loss, operative time, and patient age.^5^ It should be emphasized however that increased blood loss is not due to frequent scope removal itself, but rather intraoperative events that occur independent of scope removal. Another study by Yong et al. found that during 64 hours of observed laparoscopic surgery, just 56% of the operative time was performed with clear vision and 7% of the time was spent cleaning the lens.^4^ It is clear that frequent scope removal impedes operative efficiency, but based on available evidence, surgeons are most likely to benefit from the cleaning method that best suits one’s surgical practice.

## Conclusion

Maintaining a clear visual field during laparoscopic surgery is essential to optimize operative efficiency, and potentially patient outcomes. This study demonstrates that a novel hydrophilic scope cleaning device has comparable performance to heated anti-fogging solution and may reduce scope removals and debris events with similar cleaning time. Our narrative review describes a range of available scope cleaning strategies, from simply heating the laparoscope to innovative commercial products, that can be used to effectively maintain a clear visual field by preventing lens fogging and clearing operative debris. However, there is a paucity of clinical outcomes data pertaining to these cleaning methods. In the absence of a definitive frontrunner, surgeons are most likely to be successful with the cleaning strategy that best suits one’s surgical practice.

## References

[1] St John A Caturegli I Kubicki NS Kavic SM . The rise of minimally invasive surgery: 16 Year analysis of the progressive replacement of open surgery with laparoscopy. JSLS. 2020;24(4):e2020. doi:10.4293/JSLS.2020.00076 PMC7810432 33510568

[2] Van Deurzen DFP Mannaerts GHH Jakimowicz JJ Cuschieri A . Prevention of lens condensation in laparoscopic surgery by lens heating with a thermos flask. Surg Endosc. 2005;19(2):299-300. doi:10.1007/s00464-004-8231-4 15580442

[3] Lawrentschuk N Fleshner NE Bolton DM . Laparoscopic lens fogging: a review of etiology and methods to maintain a clear visual field. J Endourol. 2010;24(6):905-913. doi:10.1089/end.2009.0594 20370436

[4] Yong N Grange P Eldred-Evans D . Impact of laparoscopic lens contamination in operating theaters: a study on the frequency and duration of lens contamination and commonly utilized techniques to maintain clear vision. Surg Laparosc Endosc Percutan Tech. 2016;26(4):286-289. doi:10.1097/SLE.0000000000000289 27438176

[5] Abbitt D Khallouq BB Redan J . Quantifying intraoperative laparoscopic visual field opacity. JSLS. 2017;21(2):e2017. doi:10.4293/JSLS.2017.00004 PMC5444557 28584499

[6] Nabeel A Al-Sabah SK Ashrafian H . Effective cleaning of endoscopic lenses to achieve visual clarity for minimally invasive abdominopelvic surgery: a systematic review. Surg Endosc. 2022;36(4):2382-2392. doi:10.1007/s00464-021-08519-6 33963440 PMC8921162

[7] Golestani S Hill C Ali J Idelson C Rylander C Uecker J . A clean sweep: initial experience with a novel intracavity laparoscopic cleaning device. JSLS. 2022;26(4):e2022.00066. doi:10.4293/JSLS.2022.00066 PMC9840218 36721732

[8] Kitano S Tomikawa M Iso Y , et al. A safe and simple method to maintain a clear field of vision during laparoscopic cholecystectomy. Surg Endosc. 1992;6(4):197-198. doi:10.1007/BF02210882 1387738

[9] Drysch A Schmitt K Uribe B Yoon R Okhunov Z Landman J . Comparative analysis of techniques to prevent laparoscopic fogging. Minim Invasive Ther Allied Technol. 2016;25(6):319-322. doi:10.1080/13645706.2016.1203798 27384967

[10] Merkx R Muselaers C d’Ancona F , et al. Effectiveness of heated sterile water vs ResoClear® for prevention of laparoscopic lens fogging in a randomized comparative trial. J Endourol. 2018;32(1):54-58. doi:10.1089/end.2017.0683 29186976

[11] Song T Lee DH . A randomized Comparison of laparoscopic LEns defogging using Anti-fog solution, waRm saline, and chlorhexidine solution (CLEAR). Surg Endosc. 2020;34(2):940-945. doi:10.1007/s00464-019-06852-5 31139989

[12] Manning TG Papa N Perera M , et al. Laparoscopic lens fogging: solving a common surgical problem in standard and robotic laparoscopes via a scientific model. Surg Endosc. 2018;32(3):1600-1606. doi:10.1007/s00464-017-5772-x 28791559

[13] Brown JA Inocencio MD Sundaram CP . Use of a warming bath to prevent lens fogging during laparoscopy. J Endourol. 2008;22(11):2413-2414. doi:10.1089/end.2008.0212 19046081

[14] Palvia V Gonzalez AJH Vigh RS Anasti JN . A randomized controlled trial comparing laparoscopic lens defogging techniques through simulation model. Gynecol Minim Invasive Ther. 2018;7(4):156-160. doi:10.4103/GMIT.GMIT_39_18 30306034 PMC6172873

[15] Nezhat C Morozov V . A simple solution to lens fogging during robotic and laparoscopic surgery. JSLS. 2008;12(4):431. 19275865 PMC3016008

[16] Cassera MA Goers TA Spaun GO Swanström LL . Efficacy of using a novel endoscopic lens cleaning device: a prospective randomized controlled trial. Surg Innov. 2011;18(2):150-155. doi:10.1177/1553350611399297 21343172

[17] Medeon. Accessed February 20, 2024. https://www.medeonbiodesign.com/clickclean/

[18] Kinopicz . (n.d.). Floshield Air. FloShield. https://floshield.com/floshield-air

[19] Bendifallah S Salakos E Naoura I , et al. Prospective, randomized comparison of the use of FloShield Air System® versus the reference technique (water + povidone-iodine solution) during gynecologic endoscopic surgery to evaluate the operative lens vision quality. Surg Endosc. 2018;32(3):1593-1599. doi:10.1007/s00464-017-5642-6 28643058

[20] Ozgur I Liska D Valente MA Steele SR Gorgun E . Stop the smudge: a novel solution to loss of vision during laparoscopic colorectal surgery. Surg Laparosc Endosc Percutan Tech. 2022;32(5):534-536. doi:10.1097/SLE.0000000000001091 36044315

[21] Watanabe J Suwa Y Goto K , et al. Randomized controlled trial evaluating the effect of the use of a laparoscopic lens-cleaning device during laparoscopic colorectal surgery on the multidimensional workload (YCOG1903). Surg Endosc. 2023;37(6):4748-4753. doi:10.1007/s00464-023-09972-1 36894809

